# The association between NLGN4 gene variants and the incidence of autism spectrum disorders in Guilan, Iran

**DOI:** 10.1016/j.ibneur.2025.01.018

**Published:** 2025-02-08

**Authors:** Sepideh Atefrad, Aidi Yousefnejad, Niloofar Faraji, Parvaneh Keshavarz

**Affiliations:** a Department of Genetics, Cellular and Molecular Research Center, School of Medicine, Guilan University of Medical Sciences, Rasht, Iran; b Master of Genetics, Razi Clinical Research Development Unit, Guilan University of Medical Sciences, Rasht, Iran; c Gastrointestinal and Liver Diseases Research Center, Guilan University of Medical Sciences, Rasht, Iran

**Keywords:** Autism Spectrum Disorder, NLGN4, Polymorphism

## Abstract

Autism Spectrum Disorder (ASD) is a developmental disorder characterized by impaired social interaction, communication skills, and repetitive behaviours. This study aimed to investigate the association between variants of the Neuroligin-4 (NLGN4) gene (rs1882260 and rs3810688) and the incidence of ASD in North of Iran in the ASD group (n = 60) and control group (n = 60). DNA was isolated from whole blood, saliva, or hair samples. The targeted variants were genotyped using the Amplification Refractory Mutation System-Polymerase Chain Reaction (ARMS-PCR) technique. Genetic analyses were conducted using SNPAlyze ver. 8.1. Results revealed a significant difference of rs3810688 polymorphism in the NLGN4 gene in both genotypic and allelic frequency distributions between the ASD and control groups (P < 0.05). The GG genotype of rs3810688 polymorphism exhibited a significant association with an elevated risk of ASD in contrast to the CC genotype, as revealed under the co-dominant model (OR=4.2; 95 %CI, 1.25–14.05; P = 0.019). The study illustrated the possible role of rs3810688 polymorphism of NLGN4 in increasing the incidence of ASD among newborns in Guilan province. Also, the G-C haplotype was found to be a protective variant against ASD.

## Introduction

Autism Spectrum Disorder (ASD) is a developmental disability manifesting in impaired communication and social interaction that often emerges with age, impacting academic performance and involving repetitive behaviours and sensory sensitivities (Hus and Lord, 2014, Hodges et al., 2020). The prevalence of ASD has risen notably over recent decades, with its etiology attributed to a multifaceted interplay of genetic and environmental factors. prevalence was markedly elevated among children aged 6–12 years compared to those under five and those aged 13 years and above (Talantseva et al., 2023). Various environmental factors, including nutritional elements, hormonal imbalances, rubella virus infections, and cytomegaloviruses, have been implicated in ASD pathogenesis (Genovese and Butler, 2023, Keil-Stietz and Lein, 2023, Lamanna and Meldolesi, 2024).

The etiology of autism involves a significant genetic component, supported by evidence from chromosomal aberrations, rare monogenic mutations, and the cumulative effects of common genetic variants (Lamanna and Meldolesi, 2024). Studies demonstrated a strong genetic association between de novo mutations and the incidence of ASD in patients with a family history of ASD (Alonso-Gonzalez et al., 2018, Dhaliwal et al., 2021). The Neuroligin-4 (NLGN4) gene is crucial for synaptic formation and neuronal communication, encoding postsynaptic adhesion molecules necessary for synaptic signalling. Mutations in NLGN4 are linked to ASD and other neurodevelopmental disorders. Furthermore, mutations in NLGNs, which mediate synaptic function by interacting with presynaptic neurexins (NRXNs), have been identified as a genetic syndrome associated with ASD (Cast et al., 2021, Südhof, 2008).

Heterogeneity is evident concerning NLGN4, as humans possess both NLGN4X on the X chromosome and NLGN4Y on the Y chromosome, exhibiting approximately 97 % sequence similarity. These genes, forming an X-Y pair, are essentially identical (Maxeiner et al., 2020a, Lawson-Yuen et al., 2008). Mutations in the human-specific NLGN4 gene have been frequently observed in individuals diagnosed with autism and various neurodevelopmental disorders. Although the NLGN4 gene product remains relatively underexplored, it shares approximately 70 % sequence identity with other members of the neuroligin family, known to facilitate synaptic formation and function by binding to neurexin (Toya et al., 2023, Nguyen et al., 2020a).

Comprehending the genetic foundation of ASD is essential for formulating targeted interventions and therapeutic approaches. Variants rs3810688, rs5916269, and rs140700235 were identified within putative binding sites of has-miR-1293, has-miR-1324, and has-miR-5011–5p. These SNPs may influence NLGN4 expression by modifying the interaction between miRNAs and mRNA. The allele A of rs3810688 in the 3′UTR of NLGN4 exhibits a mismatch with the seed region of miR-1293 and has a modified minimum free energy value (Nguyen et al., 2020a, Liu et al., 2013). Nonetheless, the precise impact of these mutations on NLGN4's molecular properties and their consequent effects on synaptic transmission in human neurons remained poorly understood. In this regard, in the current study, we investigated the variants of the NLGN4 gene and their association with ASD among newborns in Guilan province, Iran.

## Material and methods

### Study design and sampling

This case-control study was conducted on 60 children diagnosed with ASD as a case group and 60 healthy individuals (without ASD) as a control group in Guilan province, Iran. All participants were under 18, and their parents or legal gardeners gave their written informed consent to participate in the study. All procedures performed in the study were by the ethical standards of the institutional and/or national research committee and with the 1964 Helsinki Declaration and its later amendments or comparable ethical standards. All data of patients including age, gender, habitat (urban or rural), ethnicity, family history of ASD, status of mother’s underlying diseases (hypertension and diabetes), status of parents’ smoking habits, history of previous diseases (jaundice, smallpox, and rubella), and underlying diseases attention deficit hyperactivity disorder (ADHA), brain abscess, meningitis, intellectual disabilities, epilepsy, tic disease, schizophrenia, and panic disorder) were collected. Children with chromosomal abnormalities, fragile X syndrome, tuberous sclerosis, malformation features, or any other neurological conditions suspicious of ASD were excluded from the study.

### DNA extraction

About 2 cc of peripheral blood samples were collected from the participants. In cases where children did not cooperate with collecting peripheral blood samples, saliva sampling or 10–25 uprooted hairs were considered alternatives. The DNA was extracted from all samples using the standard procedure and the Geneall® Exgene™ Genomic DNA micro® kit (Seoul, Republic of Korea).

### Genotyping

Amplification Refractory Mutation System-Polymerase Chain Reaction (ARMS-PCR technique was employed to ascertain the genotype of the samples, offering a robust means to identify point mutations (Little, 2001). This method entailed the utilization of both mutated and wild-type primers in separate reaction tubes. Amplification of the target DNA fragment in the tube containing the wild-type primer signified the absence of a point mutation at the desired locus. Conversely, amplification in the tube containing the mutated primer indicated the presence of a point mutation at the designated locus. The ARMS-PCR setup used two forward primers (mutated and wild-type) and a typical reverse primer. The distinction between the forward primers lay in the point mutation site at the 3′ end of these primers. The sequence information for relevant polymorphic sites was sourced from the NCBI database. Subsequently, primers were designed using Premer3 online and GeneRunner software (Table 1).

**Table 1:** Forward and reverse primers for NLGN4 gene variants.

**SNP**	**Forward Primer**	**Reverse Primer**	**Tm (°C)**	**PCR product length**
**rs1882260**	5′-GAAGATCATGTTCCTCGGCTCCA−3′	R1(Wild): 5′-AGGATTTGGGGGTGACTTGTTCG−3′	58	235 bp
		R2 (Mutant): 5′-AGGATTTGGGGGTGACTTGTTCT−3′		
**rs3810688**	5′-AGTACAAATTTACCCCACGG−3′	R1(Wild): 5′-GGGACAAAAACATTCCTGCTC−3′	50	154 bp
		R2 (Mutant): 5′-GGGACAAAAACATTCCTGCTG−3′		

Single nucleotide polymorphism (SNP)

Different DNA volumes were utilized in each reaction based on each sample's extracted DNA concentration. For concentrations below 50 ng/µl, 3 µl of DNA was employed, whereas concentrations ranging from 50 ng/µl to 150 ng/µl necessitated 2 µl. For concentrations between 150 ng/µl and 500 ng/µl, 1 µl of DNA was used, while for concentrations exceeding 500 ng/µl, 0.5 µl was employed. Dilution of DNA was performed for exceptionally high concentrations. The volume of H_2_O utilized in each reaction varied depending on the DNA concentration. PCR process included 5 min for one cycle of first denaturation at 95°c; 90-sec amplification for 32 cycles, in which 30 sec was for denaturation at 95°c and 30 sec for annealing rs1882260 in 58°c and rs3810688 in 50°c, and 30 sec for extension in 72°c; 5 min for final extension of 1 cycle in 72°c; and holding at 4°c. The quantity and quality of DNAs were confirmed by NanoDrop spectrophotometer and agarose gel electrophoresis (agarose gel 0.8 %), respectively (Miller et al., 1988, Lee et al., 2012, García-Alegría et al., 2020).

### Statistical analysis

The frequency of demographical and clinical data of the participants were reported as numbers and percentages. Statistical analyses of linkage disequilibrium (LD), the pairwise delta (correlation co efﬁcient), Hardy Weinberg Equilibrium (HWE), and haplotype frequency with CI 95 % were analyzed via SNPAlyze software (Dynacom.8.0, Tokyo, Japan), MedCalc and SPSS version 20, with a significance level < 0.05. To ascertain the association of the NLGN4 gene, univariate analysis of covariance (ANCOVA) was employed. The regression results were reported by odds ratio (OR) with 95 % confidence interval (CI).

## Results

### Frequency of demographical and clinical data of participants

The findings highlighted predominantly of males in both studies group. A significant proportion of children with ASD resided in rural areas (91.7 %), a demographic pattern that might reflect environmental or healthcare access factors influencing diagnosis. Notably, the ASD group exhibited a higher prevalence of maternal comorbidities and childhood diseases such as panic disorder (21.7 %) and ADHD (18.3 %), underlining the complex interplay between genetic predispositions and other risk factors (Table 2).

**Table 2:** Demographical and clinical data of children with Autism Spectrum Disorder (ASD).

**Variables**			**Children with ASD** n (%)	**Children without ASD** n (%)
**Gender**	Male		46 (76.7)	40 (66.7)
	Female		14 (23.3)	20 (34.3)
**Smoking status of parents**	Never	Mother	39 (65.0)	17 (28.3)
		Father	32 (53.3)	53 (88.3)
	Sometimes	Mother	8 (13.3)	10 (16.6)
		Father	9 (15.0)	7 (11.6)
	Always	Mother	4 (6.7)	3 (5.0)
		Father	9 (15.0)	24 (40.0)
**Habitat**	Rural		55 (91.7)	-
	Urban		5 (8.3)	-
**Childhood disease**	Smallpox		5 (8.3)	-
	Jaundice		9 (15.0)	10 (16.6)
	Rubella,		-	-
	Intellectual disabilities		10 (16.6)	-
	Epilepsy		4 (6.7)	1 (1.7)
	Brain abscess		-	-
	ADHD		11 (18.3)	1 (1.7)
	Schizophrenia		-	-
	Panic disorder		13 (21.7)	-
**Underlying disease of the mother**	Hypertension		6 (10.0)	-
	Diabetes		2 (3.3)	-
**Family background**			12 (20.0)	9 (15.0)

*The differences between the total number of cases and controls are due to the data that do not apply to variables

Attention deficit hyperactivity disorder (ADHD)

### Allele frequency of NLGN4 gene polymorphisms

**Fig. 1:**
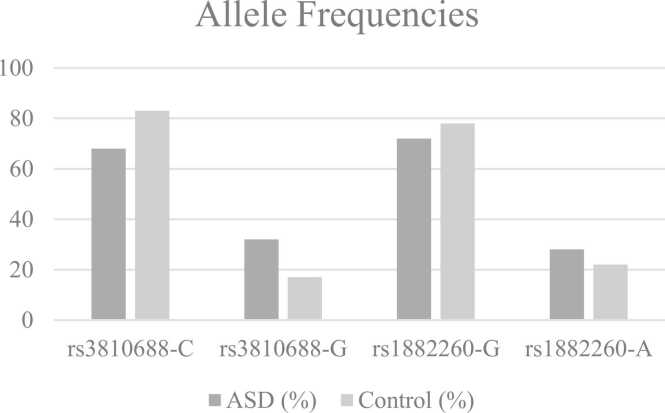
Allele Frequencies: the distribution of alleles for rs3810688 and rs1882260 between the ASD and control groups. The significant difference in the C allele of rs3810688 is evident (P = 0.004).

The G allele frequency of rs1882260 polymorphism was 72.0 % and 78.0 % in the ASD and control groups, respectively. Allelic frequency distributions between the ASD and control groups illustrated no statistically significant difference between the patient and control groups (P > 0.05). The allele frequency of the rs3810688 variant showed an essential difference between ASD and control groups, with the C allele frequency notably lower in the ASD group (68 % vs. 83 %, P < 0.05) (Fig. 1). This suggests a potential protective role of the C allele. In contrast, the rs1882260 variant did not display significant differences in allele frequencies or genotype distributions between the groups, indicating its limited contribution to ASD risk in this population.

Genotype analysis of rs3810688 revealed a significant association under the co-dominant and recessive models. Specifically, the GG genotype was identified as a risk genotype, with an odds ratio (OR) of 4.2 under the co-dominant model (P = 0.019) and 3.87 under the recessive model (P = 0.020). These findings underscore the role of rs3810688 polymorphism in increasing ASD susceptibility (Table 3).

**Table 3:** Allelic and genotypic frequencies of the NLGN4 gene among children with and without Autism Spectrum Disorder (ASD).

Variants	ASD n = 60 (%)	Control n = 60 (%)	χ^2^ test	*P value*
rs1882260				
G A	87 (72.0) 33 (28.0)	93 (78.0) 27 (22.0)	0.79	0.370
rs1882260				
GG GA AA	37 (61.6) 19 (31.7) 4 (6.7)	33 (55) 21 (35) 6 (10)	0.72	0.690
rs3810688				
C G	81 (68.0) 39 (32.0)	100 (80.0) 17 (20.0)	8.07	0.004
rs3810688				
CC CG GG	34 (56.6) 13 (21.7) 13 (21.7)	44 (73.3) 12 (20.0) 4 (6.7)	6.08	0.047
Codominant				
CC CG GG	34 (56.6) 13 (21.7) 13 (21.7)	44 (73.3) 12 (20.0) 4 (6.7)	Ref 1.4 (0.56–3.45) 4.2 (1.25–14.05)	- 0.460 0.019
Dominant				
CC CG+GG	34 (56.7) 26 (43.3)	44 (73.3) 16 (26.7)	Ref 2.1 (0.97–4.52)	- 0.050
Recessive				
CC+CG GG	47 (70.3) 13 (21.7)	56 (93.3) 4 (6.7)	Ref 3.87 (1.18–12.67)	- 0.020

Results of Pearson (χ2) tests and bidirectional logistic regression using SPSS software version 20

**Table 4:** Haplotype analysis for studied polymorphic sites in the NLGN4 gene among children with and without Autism Spectrum Disorder (ASD).

**Number of holotypes**	**Haplotype** rs1882260- rs3810688	**Total frequency**	**Control**	**ASD**	**Permutation p-value**
1	G-C	0.581	0.672	0.312	< 0.001
2	G-G	0.240	0.197	0.387	0.073
3	A-C	0.140	0.127	0.212	0.195
4	A-G	0.038	2.621-E	0.087	0.087

(ASD) (1 =Major Allele Homozyote, 2 =Heterozygote, 3 = Minor Allele Homozygote)

### Genotype distribution of NLGN4 gene polymorphisms

HWE was established between groups in terms of genotype distributions of both polymorphisms (rs1882260 and rs3810688) in the SAD group (P = 0.666 and P = 0.184), respectively, and in the control group (P = 0.456 and P = 0.375), respectively. At the polymorphic locus rs1882260 of the NLGN4 gene, three distinct genotypic profiles (GG, GA, and AA) were identified within the ASD and control groups. The GG genotype was more prevalent, 61.6 % and 55 % in the ASD and control groups, respectively. No significant disparity was discerned in the genotypic frequency distribution between the patient and control groups (P > 0.05). For the polymorphic locus rs3810688 within the NLGN4 gene, CC, CG, and GG genotypes were reported, in which the frequency of CC genotype was higher in both ASD and control groups with the frequency of 56.6 % and in the ASD group and 73.3 % in control group. A significant difference in genotypic frequency distribution between ASD and control groups was observed (P < 0.05) (Table 3). Based on the LD and r^2^ analysis, the two rs1882260 and rs3810688 polymorphisms were not strong in LD.

### Genotyping distribution according to inheritance pattern in study polymorphisms

Genotyping distribution demonstrated a significant association between three genotypes of rs3816088 polymorphism and the incidence of ASD under the co-dominant inheritance model (OR=4.2; 95 %CI, 1.25–14.05; P = 0.019). The significance of the GG genotype as a risk genotype for ASD was also evident in the recessive model (OR=3.87; 95 %CI, 1.18–12.67; P = 0.020) (Table 3).

### Haplotype association of NLGN4 gene Polymorphisms and ASD disease

Haplotype analysis further illuminated the genetic associations. The G-C haplotype (rs1882260-rs3810688) emerged as a protective factor against ASD (P < 0.001), with its frequency significantly lower in the ASD group (31.2 %) compared to controls (67.2 %). Conversely, the G-G haplotype exhibited a higher prevalence in the ASD group, suggesting a potential risk factor.

### Interpretation and implications

These results reinforce the importance of rs3810688 in ASD pathogenesis, particularly its GG genotype and associated haplotypes. The findings align with previous studies emphasizing the role of NLGN4 variants in synaptic function and ASD etiology. However, the absence of significant associations with rs1882260 highlights the locus-specific impact of genetic variants.

Given the heterogeneity of ASD, integrating these genetic insights with broader genomic and environmental data is essential. Future research should aim to validate these findings in more extensive, diverse cohorts and explore the functional implications of rs3810688 polymorphisms on NLGN4 expression and neuronal connectivity.

## Discussion

ASD presents with social communication deficits and repetitive behaviors, and genetic factors play a significant role in ASD development, evidenced by studies identifying various genetic contributors, from rare to common variants (Havdahl et al., 2021, Zhang et al., 2021). Among these, NLGN4 stands out due to its synaptic role and association with neurodevelopmental disorders. Investigating the link between NLGN4 gene variants and ASD is crucial for understanding ASD's genetic basis and potential therapeutic strategies (Zhang et al., 2018, Upadhyay et al., 2021). While NLGN4 mutations are rare in ASD cases, their influence on synaptic function offers valuable insights into ASD pathogenesis. Beyond the coding sequence, investigating the regulatory region and copy number variations of NLGN4 is crucial in ASD research, as they could modulate NLGN4 expression levels, further elucidating the disorder's underlying mechanisms (Nakanishi et al., 2017, Nguyen et al., 2020b).

A study by Südhof highlighted how mutations in the NLGN4 gene disrupt synaptic function, contributing to neurodevelopmental and cognitive disorders, including ASD. Specifically, it emphasized the mechanistic basis of neuroligin-neurexin interactions influencing synaptic transmission, providing a biochemical framework to understand how polymorphisms like rs3810688 may impact ASD susceptibility (Südhof, 2008). Nguyen et al. demonstrated how mutations in NLGN4X and NLGN4Y genes, such as frameshifts or missense variants, impair synaptic signalling. Their findings supported that structural alterations in neuroligin proteins could lead to the neuronal dysfunction observed in ASD, highlighted that coding and non-coding mutations could have significant neurodevelopmental consequences (Nguyen et al., 2020a).

A study by T. P. Cast et al. found that altered glycosylation impairs the structural integrity of synapses, particularly excitatory ones, which may account for ASD-related behavioral phenotypes. This aligns with the current study's findings by illustrating how the rs3810688 variant could alter synaptic functionality, increasing ASD risk (Cast et al., 2021). Maxeiner et al. demonstrated that human-specific adaptations in NLGN4 might underlie its significant association with neurodevelopmental disorders like ASD (Maxeiner et al., 2020b). Another study highlighted that rural residency is a key demographic factor influencing ASD prevalence. By integrating these external influences with genetic data, the study emphasized the complex, multifactorial nature of ASD development (Chen et al., 2014).

The findings of a study by Zhou et al. on the protective effects of specific haplotypes, such as the G-C haplotype, resonate with the current study’s conclusions (Zhou et al., 2022). Liu et al. explored six single nucleotide polymorphisms (SNPs) in NLGN4 within a Chinese population and found no significant association with ASD. They reported that population-specific factors, such as genetic background and environmental exposures, could explain the discrepancies between their results and those of other studies (Liu et al., 2013). On the contrary, we found a strong association for rs3810688 in the Iranian cohort

A study by Landini et al. identified several variants in promoter and untranslated regions (UTRs) that could influence mRNA stability and protein translation. These findings align with the current study's suggestion that non-coding haplotypes, such as rs3810688, may have regulatory impacts on NLGN4 expression and contribute to ASD risk (Landini et al., 2016). Rodgaard et al. noted that comorbidities, such as ADHD and panic disorders, were more common among males, consistent with the current study's demographic data. They also suggested that genetic factors, including sex-linked variants like NLGN4X, play a critical role in this gender disparity (Rødgaard et al., 2021). Kinney et al. explored the role of prenatal environmental stressors, such as maternal health conditions and nutritional deficiencies, in modulating ASD risk. They reported that these stressors could increase the sensitivity of the developing brain, amplifying the effects of genetic predispositions. This complements the current study’s observation of maternal comorbidities in the ASD group, reinforcing the interaction between genetic and environmental factors in ASD etiology (Kinney et al., 2008). Nakanishi et al. analyzed rare variants in neuroligin genes, including NLGN4, and their impact on synaptic plasticity, demonstratinh how mutations could disrupt excitatory/inhibitory balance, consistent with the current study’s findings on rs3810688’s role in ASD risk (Nakanishi et al., 2017).

In light of the heterogeneity observed in ASD regarding symptoms, severity, and comorbidities, it is plausible that multiple genes, along with epigenetic and environmental factors, contribute to ASD susceptibility. Consequently, attributing a significant role in ASD predisposition to a single genetic variant or haplotype, even within regulatory regions or candidate genes, appears improbable. Therefore, conducting additional, comprehensive functional and genetic investigations integrating SNP profiles within a larger ASD cohort is imperative to elucidate further the involvement of neuroligins and genetic variants in ASD etiology.

## Conclusion

Our results demonstrated a significant association between the rs3810688 polymorphism of the NLGN4 gene and the risk of ASD in our study population. Specifically, the G-C haplotype was identified as a protective variant against ASD, as evidenced by its lower frequency in individuals with ASD compared to controls.

## Ethics Statement

All procedures performed in studies involving human participants were by the ethical standards of the institutional and/or national research committee and with the 1964 Helsinki declaration and its later amendments or comparable ethical standards.

## Funding

No Funding.

## Consent to Participate

Informed consent was obtained from all individual participants included in the study.

## Consent for Publication

The authors affirm that human research participants provided informed consent for the study's publication.

## CRediT authorship contribution statement

**Atefrad Sepideh:** Writing – review & editing, Writing – original draft, Methodology, Investigation, Data curation. **Yousefnejad Aidi:** Investigation, Data curation. **Keshavarz Parvaneh:** Supervision, Resources, Project administration, Methodology, Formal analysis, Conceptualization. **Faraji Niloofar:** Writing – review & editing, Writing – original draft.

## Declaration of Competing Interest

There is no conflict of interest to declare.

## Data Availability

Data will be made available on request.
